# Reconciling profiles of reward-seeking versus reward-restricted behaviours in frontotemporal dementia

**DOI:** 10.1093/braincomms/fcad045

**Published:** 2023-03-01

**Authors:** Kristina Horne, Muireann Irish

**Affiliations:** School of Psychology, The University of Sydney, Sydney, Australia; Brain and Mind Centre, The University of Sydney, Sydney, Australia; School of Psychology, The University of Sydney, Sydney, Australia; Brain and Mind Centre, The University of Sydney, Sydney, Australia

## Abstract

This scientific commentary refers to ‘The architecture of abnormal reward behaviour in dementia: multimodal hedonic phenotypes and brain substrate’, by Chokesuwattanaskul *et al.* (https://doi.org/10.1093/braincomms/fcad027).

**This scientific commentary refers to ‘The architecture of abnormal reward behaviour in dementia: multimodal hedonic phenotypes and brain substrate’, by Chokesuwattanaskul *et al.* (https://doi.org/10.1093/braincomms/fcad027).**

Alterations in reward processing are well documented in frontotemporal dementia (FTD), reflecting pathological changes in the frontal and striatal regions of the brain. A recent study by Chokesuwattanaskul *et al*.^1^ offers compelling new insights into the multidimensional nature of hedonic disturbances in FTD and provides a new framework by which we might reconcile seemingly conflicting profiles of reward-seeking versus reward-restricted behaviours in these syndromes.

Using a transdiagnostic approach, the authors employed a semi-structured questionnaire to assess changes in responsiveness to primary (e.g. food and sexual behaviour) and non-primary (e.g. music, religion, art and colours) reward types. For each item, carers identified changes in relevant behaviours and the dominant direction of this change (i.e. increased or decreased). Alterations in reward behaviours were most prominent in behavioural variant (bvFTD; 96%), and semantic variant (svPPA; 86%) patients, with an ‘eating-predominant’ phenotype emerging as the most common reward cluster, resonating with previous studies.^2,3^ Interestingly, the ‘reward-seeking’ phenotype occurred most frequently in svPPA, while the ‘reward-restricted’ phenotype was most prevalent in bvFTD. In contrast, most patients with Alzheimer’s disease, logopenic and non-fluent variants of Primary Progressive Aphasia, displayed a ‘control-like’ phenotype, suggesting minimal change in responsiveness to rewards.

Multiple correspondence analysis identified two principal reward factors, namely, a ‘gating factor’ indicating the presence of *any* change in responsiveness regardless of the goal or direction and a ‘modulatory’ factor that specifies the tuning of behaviours to prioritize goals and minimize adverse outcomes (i.e. the direction of the behaviour). The authors propose that these two factors operate orthogonally to determine an individual’s overall reward phenotype. Finally, voxel-based morphometry analyses related these two principal factors to underlying patterns of grey matter atrophy. A bilateral anterior network comprising the anterior cingulate gyrus, bilateral temporal poles, right fusiform gyrus and right middle frontal gyrus was implicated in the ‘gating factor’. No significant associations were found between grey matter intensity and the second ‘modulatory factor’, likely reflecting the bidirectional nature of this dimension.

Changes in hedonic capacity have typically been conceptualized within effort-based decision-making for reward frameworks, in which goal-directed (i.e. reward-seeking) behaviour can be deconstructed into a set of fundamental subcomponents.^4^ Applying this framework, we might liken the ‘modulatory’ factor uncovered by Chokesuwattanaskul *et al*. to anticipatory aspects of the hedonic experience (i.e. ‘wanting’), which influence whether the individual shows increased or decreased interest in a particular behaviour. In contrast, the principal ‘gating’ factor, which indexes *any* change in hedonic function, is not accommodated as readily by this framework and could reflect an impaired integration of reward and effort signals in the decision-making phase, misrepresentation of reward values of behaviours due to poor reinforcement learning and/or changes in the hedonic (i.e. consummatory) experience itself.

Crucially, the phenotypes elucidated by Chokesuwattanaskul *et al*.^1^ enable us to move away from discrete categorical labels to explore how abnormal reward behaviours in FTD might exist within a multidimensional space. Understanding graded alterations across dimensions of hedonic function provides a clinically useful framework to reconcile heterogeneity at the individual case level. For instance, most participants in the ‘reward-restricted’ phenotype demonstrated an increased responsiveness to colour, alongside a decreased responsiveness to music, and/or a reduced libido. Similarly, within the ‘eating-predominant’ reward phenotype, patients demonstrated increased appetite, preference for sweet foods and responsiveness to music, in the context of reduced libido and responsiveness to art. These findings highlight that individuals within the same diagnostic category can simultaneously display diminished interest or engagement towards some reward types alongside excessive or preferential pursuit of others.

At first glance, the co-occurrence of increased and decreased reward responsiveness in a population characterized by a profound loss of motivation might seem difficult to reconcile. To date, the vast majority of studies exploring motivational changes in FTD suggest striking reductions in goal-directed behaviour as well as a marked attenuation in the capacity for pleasure (i.e. anhedonia).^5,6^ An increased and, at times obsessive, pursuit of new interests has been documented in patients with the right temporal variant of FTD (rtvFTD).^7^ Indeed, a recently published framework of rtvFTD suggests that compulsive behaviours (40% of patients) co-occur with apathy (55% of patients) as common initial symptoms, while dietary changes and hyperorality emerge later in the disease course.^7^ Reported compulsive behaviours included clock watching, ritualistic preoccupations (e.g. dressing each day of the week in a different colour) and in one patient, repeatedly driving over an hour to a specific shop to buy items for a small discount.

In terms of underlying mechanisms, a failure to inhibit reward pursuit has been put forward as one candidate for obsessive behaviours.^3^ However, if this were the sole contributing mechanism, we would predict a profile of widespread and undifferentiated reward-seeking in FTD, which is not the case. Rather, FTD patients often present with a relatively circumscribed but heightened reward-seeking behaviour in the context of a generalized reduction in motivation^7^ and hedonic tone.^5^

How then might we reconcile the co-occurrence of amplified and attenuated reward behaviours within the same clinical phenotype? Theoretical models of reward processing offer some important starting points. For instance, dampened reward sensitivity may increase the need to repeat or intensify a behaviour to pass a heightened threshold for consummatory pleasure. Furthermore, a breakdown in reward learning (updating) might give rise to repetitive behaviours, even when there is no positive outcome or the outcome itself is aversive.

The targeted nature of reward-seeking behaviour in rtvFTD suggests that the stimulus itself plays an important role. Given the canonical semantic impairments in this syndrome, it may be that individuals lose their knowledge of the inherent reward value of some behaviours or stimuli, narrowing the pool of rewarding experiences from which the patient can reliably sample. As the conceptual knowledge base progressively deteriorates, patients increasingly rely on familiar recent experiences as templates for future behaviour, leading to an increasingly inflexible and rigid style of interacting with the world.^8^ These recent experiences may potentiate a repetitive cycle of inflexible reward-seeking behaviours directed to one or two specific activities that remain salient or meaningful to the individual. This cycle may also be exacerbated by symptoms of apathy and anhedonia, as patients lack the motivation to expend effort in the decision-making phase to consider alternative behaviours. This hypothetical iterative cycle is depicted in Fig. 1.

**Figure 1:**
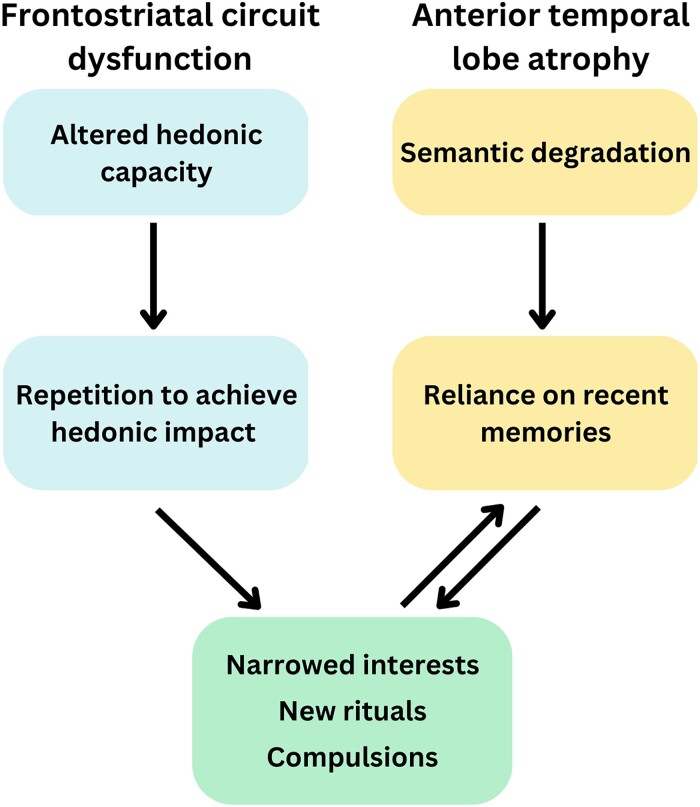
**Dual pathways underlying the emergence of repetitive behaviours and restricted interests in FTD.** We propose that frontostriatal dysfunction in FTD disrupts a *primary hedonic pathway* that diminishes the fundamental capacity to experience pleasure (i.e. anhedonia). This dampening of pleasure is posited to result in a narrowing of interests, as formerly pleasurable activities no longer bring enjoyment. However, some activities might confer residual feelings of pleasure if a minimum threshold is surpassed, leading to repetitive behaviours in the pursuit of reward. Temporal lobe dysfunction can potentiate repetitive behaviours via a *secondary semantic pathway*, by which the representation of general world knowledge regarding activities, objects and their corresponding reward properties is disrupted. With increasing semantic deterioration, recent events become the dominant template for future behaviours, leading to a cycling of intensified behaviours towards specific targets, most pronounced in semantic variants of FTD.

Evidence of potentiated reward-seeking behaviours in other temporal lobe pathologies supports our proposal. For instance, temporal lobe epilepsy has long been associated with ‘Geschwind Syndrome’, a cluster of symptoms including hyper-religiosity, hyper-graphia and hyposexuality, most common in right lateralized presentations.^9^ Indeed, some case reports have proposed the Geschwind cluster of symptoms as a salient early feature of the rtvFTD syndrome.^9^ Lesion studies further indicate obsessive compulsions, Geschwind syndrome and amusia in patients with right temporal lobe stroke.^10^ Taken together, these findings offer converging evidence in support of the right temporal lobe as a key node in hedonic processing (see also Shaw *et al*.^5^). Moreover, these previous studies highlight how temporal lobe damage might give rise to intensified behaviours directed towards increasingly specific targets at the expense of other, more adaptive, behaviours.

From a clinical perspective, improved characterization of reward-processing phenotypes in FTD may help caregivers to understand and reconcile seemingly conflicting behavioural changes. For example, while FTD patients may seem apathetic in general, their pursuit of new and sometimes unusual interests may make carers feel that such patients are oppositional or defiant. Effective communication of these symptoms, and their origins, may alleviate some of the stress and burden associated with FTD by helping carers to attribute these behaviours to the disease rather than the person. As such, the paper by Chokesuwattanaskul *et al*.^1^ provides an important foundation to advance our understanding of aberrant reward-processing trajectories in FTD with a view to developing targeted interventions to improve well-being.
